# Preface to Clinical Review

**Published:** 1831-01-01

**Authors:** 


### CLINICAL REVIEW,

OR,

### HOSPITAL REPORTER;

WITH

### CRITICAL COMMENTARIES.

### VIII.

For some years past the impulse to deliver clinical lectures at our great hospitals has been gradually increasing; and the great wonder is, that the example of the Royal Infirmary of Edinburgh, did not long ago render the measure universal. An apparently trifling regulation in the last curriculum of the Court of Examiners of the Apothecaries' Company,—a regulation that seems to have "dropped in," as if by mere accident, upon the train of the code, will prove a greater stimulus to the delivery of clinical lectures than all the declamations, admonitions, and reproaches that have issued from the medical press.

By this regulation, a bonus or premium is not merely held out to those hospitals and infirmaries, metropolitan and provincial, which shall furnish regular clinical instruction in the shape of commentaries at the bed-side of sickness—but, in fact, a ban is placed over the institution that fails to comply. The *additional attendance of three months* in an hospital where no clinical lectures are delivered, is tantamount to a sequestration of the school; for pupils will flock to those establishments which present the greatest quantum of knowledge with the least expenditure of time and money.

But the benefits resulting from clinical lectures will extend very far indeed beyond the mere communication of practical tuition to the class surrounding the lecturer. The teacher himself must now look about him. He cannot now walk the wards, and prescribe in the jog-trot routine of former days. He cannot descant on diseases and their remedies, without carefully observing the history, the phenomena, and the events of the cases themselves—cases that are before the eyes of the class, and which cannot be blinked or distorted to suit the views of the preceptor.

The clinical lecturers have yet another monitor before their eyes—a monitor that combines the double action of spur and rein—the Press. The nimble-fingered gentry who now pace the wards of hospitals, will not allow a single syllable that falls from the oracle of the day, to be wasted on the desert air. Every observation will be wafted on the four winds to the four quarters of the globe—there to be weighed and scrutinized, not by raw pupils, who are too ready "*jurare in verbo magistri*," but by practical critics, who can appreciate the sterling metal, and separate it from the dross—and who consequently can estimate the merits of the different labourers in this productive field of medical science.

To form a regular and periodical reunion or concentration of the numerous disjointed facts and observations which may turn up in our own and in foreign hospitals, a large portion of our PERISCOPE, under the head of CLINICAL REVIEW, has been, for some time past, dedicated; but the present state of things induces us still farther to extend the limits, augment the labours, and perfect the organization of the department in question. We confidently appeal to our past efforts, on this point —and we refer our readers to the present number of the Journal, for a specimen of the manner in which our CLINICAL REVIEW is to be conducted in future.

Clinical lectures and hospital reports are as susceptible of analysis, and as open to criticism as any other species of medical literature. In our analyses, we shall pursue our usual system of brevity; carefully winnowing the grain from the chaff—the useful facts from the superfluous verbiage. In our strictures we shall be influenced solely by practical utility—and never by captious criticism. Extempore lectures in the wards of an hospital, should never be rigidly scrutinized, in respect to manner or language—first, because they are delivered, *viva voce*, and without the advantages of study and correction —secondly, because the lecturer may be misunderstood, or (unintentionally) misrepresented by the reporter. But the inferences drawn from facts, and the soundness or unsoundness of practice, are fair objects for commentary—and we shall never appeal to the tribunal of the public, without fairly stating the opinions and practices which we criticise, and the grounds for our dissent—without acerbity of language, or personal bias.

The system of book-making has long been ridiculous, even to a proverb, both in this country and on the Continent. The same system has crept into hospital reporting on both sides of the channel, though apparently from different motives or inducements. There is no good without its attendant evil. Where reports are paid by the line or page, there is a premium offered on the adulteration of facts with words, and hence the tedious, the useless, the detrimental detail, by which the essential points of a case are buried in verbiage, and confounded among the rubbish of superfluous garnish.

It must be confessed that our continental reporters first set the example of this interminable tautology and circumlocution, and still far outstrip their imitators on this side of the channel. The French reporter subjoins his name, and glories in shewing his powers of spinning out a description. Thus, if a man in health receives a stab in the chest, which penetrates the heart, and kills on the spot, the reporter commences with a minute description of the patient's complexion, the colour of his hair, the figure of his nails, the degree of corpulence, &c. Then every organ and tissue of the body is carefully dissected and minutely described, so that the organ which received the mortal impression receives not a whit more attention than any one of the viscera not implicated in the cause of death ! ! Our readers must often have perceived of late that our youthful reporters and practitioners, in this country, are rapidly imitating the Parisian prolixity, and bid fair to exceed them in this acquirement, in the course of a very few years. It will be our study and our task to dissect away from these bloated reports, all superfluous and unseemly garniture —and, as a matter of curiosity, we shall occasionally exhibit, in contiguous columns, the licked and the unlicked bears. These comparisons will illustrate the utility—almost the necessity, of a manufactory for refining the rough materials of medical literature, and casting the purified metal in moulds that may be more sightly to the eye, and more adapted for circulation. An illustration or two, taken at random, will place the utility of our proposal in a clearer point of view than many pages of argument.

